# Sleep quality and associated factors among university students in Africa: a systematic review and meta-analysis study

**DOI:** 10.3389/fpsyt.2024.1370757

**Published:** 2024-03-11

**Authors:** Girum Nakie, Girmaw Medfu Takelle, Gidey Rtbey, Fantahun Andualem, Techilo Tinsae, Mulat Awoke Kassa, Gebresilassie Tadesse, Setegn Fentahun, Yilkal Abebaw Wassie, Tesfaye Segon, Getasew Kibralew, Mamaru Melkam

**Affiliations:** ^1^ Department of Psychiatry, College of Medicine and Health Science, University of Gondar, Gondar, Ethiopia; ^2^ Department of Nursing, College of Health Science, Woldia University, Woldia, Ethiopia; ^3^ Department of Psychiatry, School of Medicine, College of Medicine and Health Sciences, University of Gondar, Gondar, Ethiopia; ^4^ Department of Medical Nursing, School of Nursing, College of Medicine and Health Science, University of Gondar, Gondar, Ethiopia; ^5^ Department of Psychiatry, College of Health Science, Mettu University, Mettu, Ethiopia

**Keywords:** sleep quality, university students, systematic review, meta-analysis, Africa

## Abstract

**Background:**

Poor sleep quality significantly impacts academic performance in university students. However, inconsistent and inconclusive results were found in a study on sleep among university students in several African nations. Therefore, this study aimed to estimate the pooled prevalence and associated factors of poor sleep quality among university students in Africa.

**Methods:**

The databases PubMed, Scopus, Cochrane Library, Science Direct, African Journal Online, and Google Scholar were searched to identify articles. A total of 35 primary articles from 11 African countries were assessed and included in this systematic review and meta-analysis. Data were extracted by using a Microsoft Excel spreadsheet and exported to STATA version 14 for analysis. The I^2^ test was used to assess the statistical heterogeneity. A random effect meta-analysis model was employed with 95% confidence intervals. Funnel plots analysis and Egger regression tests were used to check the presence of publication bias. A subgroup analysis and a sensitivity analysis were done.

**Results:**

A total of 16,275 study participants from 35 studies were included in this meta-analysis and systematic review. The overall pooled prevalence of poor sleep quality among university students in Africa was 63.31% (95% CI: 56.91-65.71) I^2 = ^97.2. The subgroup analysis shows that the combined prevalence of poor sleep quality in East, North, West, and South Africa were 61.31 (95% CI: 56.91-65.71), 62.23 (95% CI: 54.07-70.39), 54.43 (95% CI: 47.39-61.48), and 69.59 (95% CI: 50.39-88.80) respectively. Being stressed (AOR= 2.39; 95% CI: 1.63 to 3.51), second academic year (AOR= 3.10; 95% CI: 2.30 to 4.19), use of the electronic device at bedtime (AOR= 3.97 95% CI: 2.38 to 6.61)) and having a comorbid chronic illness (AOR = 2.71; 95% CI: 1.08, 6.82) were factors significantly associated with poor sleep quality.

**Conclusion:**

This study shows that there is a high prevalence of poor sleep quality among university students in Africa. Being stressed, in the second year, using electronic devices at bedtime, and having chronic illness were factors associated with poor sleep quality. Therefore, addressing contributing factors and implementing routine screenings are essential to reduce the burden of poor sleep quality.

**Systematic Review Registration:**

https://www.crd.york.ac.uk/prospero/, identifier CRD42023493140.

## Introduction

Sleep is a naturally recurring physiological process that is important for psychological, physical, and emotional well-being (1). Sleep is also an important role in cognitive functions like judgment and memory consolidation, and vital for academic performance (2). Sleep quality is a combination of quantitative and qualitative aspects, including duration and subjective feeling of restfulness upon awakening (3). Africa is a continent that is bounded by the Mediterranean Sea to the north, the Indian and Atlantic Oceans to the east, the Atlantic Ocean to the west, and the confluence of the two oceans to the south (4). According to recent studies, non-communicable illnesses (NCDs), such as sleep disorders, are becoming more common at an alarming rate across Africa, placing a strain on healthcare resources in addition to communicable diseases (5). Due to factors including urbanization, socioeconomic development, and globalization, NCDs are becoming more common among young Africans. Taking care of these risk factors in today’s youth can drastically change how NCDs are expected to develop in Africa (5–8). Nowadays, the majority of young individuals sleep for shorter periods of time than what is advised by science (9). Good sleep quality at night facilitates the brain’s physiological repair processes and nerve cell growth. Regular engagement in these processes enhances an individual’s memory and learning capacity (10, 11). Due to a lack of resources for education, young adults in Africa typically perform worse academically, especially if they live in rural areas (12). However, African students with lesser academic standing may experience sleep disturbances as a result, particularly if they attempt to find work and their grades fall short of most employer imposed requirements (13).

Poor sleep quality is a common problem for university students, and it has a detrimental effect on their academic performance (14, 15). Compared to the general population, university students had a twofold higher prevalence of poor quality of sleep (16). This is brought on by the change from high school to college, the lessening of the role of parents, and higher expectations for academic achievement (17, 18). Poor sleep quality was found to be highly prevalent among medical students 55.64% according to a global meta-analysis study (19). A cross-sectional study was carried out in seven countries the Dominican Republic, Egypt, Guyana, India, Mexico, Pakistan, and Sudan, and the prevalence of sleep quality among university students was found to be 73.5% (20). These rates are greater than those of the general population (21, 22). A complicated interaction between genetics, academic load, technology, environmental conditions, and comorbidities is blamed for this problem. In general, it has been noted that a sizable percentage of students have trouble sleeping, which may be connected to stress from their studies (22, 23).

Poor sleep quality among university students can lead to mental and physical health issues (24). Students who have poor quality of sleep typically report common mental health issues, which many will probably need to get support for in order to successfully resolve. Poor quality of sleep has an impact on several aspects of behavior, including executive function, hormone balance, emotional control, and attentiveness. Numerous studies that involved depriving small groups of healthy individuals of all sleep for one or two nights revealed a wide range of specific abnormalities (24–26). Among the findings in the field of emotional regulation is a rise in psychopathology symptoms, like depression (27–31), anxiety (28, 29), and stress (32–34) including substance use (18, 35). Sleep is also crucial for learning and memory processes. Nonetheless, there is still much to learn about the specifics of the connection between sleep and memory formation. According to the dual process theory, declarative memory may require non-REM sleep, and procedural memory may require rapid eye movement (REM) sleep. This is because different forms of memory are associated with different sleep states (36, 37). Therefore, poor sleep can impair attention, concentration, and memory and all this leads to poor academic performance (2, 36, 38, 39). It also leads to metabolic, hormonal, and immunologic effects, causing immune suppression (40–42). Undergraduate students often experience poor sleep quality due to various factors, including irregular sleep schedules fatigue, and co-morbid chronic illness (43, 44). Sleep disturbances can worsen a person’s quality of life in addition to contributing to the early onset of chronic diseases (45). Factors such as internet addiction, substance use, depression, stress, and poor academic performance (20, 35, 46–48) are associated with poor sleep quality. New social and academic environments, reduced parental supervision, and increased academic demands contribute to poor sleep quality among this population (18, 22, 49).

Poor sleep quality among college students in African countries is a significant issue, with mixed findings across studies. It is crucial to investigate patterns of poor sleep quality to create efficient interventions and reduce the likelihood of the detrimental effects associated with poor sleep quality, such as school dropout, poor academic performance, suicide, burnout, depression, and anxiety. To date, no meta-analysis, and systematic review has been carried out to investigate the prevalence of poor sleep quality among this population. To better understand the prevalence and associated factors of poor sleep quality among university students in African countries, we did a thorough meta-analysis and systematic review study.

## Research questions

- What is the estimated pooled prevalence of poor sleep quality among university students in Africa?
- What are the associated factors for poor sleep quality among university students in Africa?

## Materials and methods

The current meta-analysis and systematic review was registered (ID CRD42023493140) in the Prospective Register of Systemic Review (PROSPERO). Our search strategy and selection of publication for the review were conducted according to the (PRISMA 2020) guideline (50) (**Additional File 1**).

### Searching strategy

This study was conducted to determine the sleep quality and associated factors among university students in Africa. A search of published articles was found by using the following databases: EMBASE, PubMed, African Journals Online, Psychiatry Online, Scopus, World Health Organization (WHO) reports, Cochrane Library, and other gray literature from Google. A search strategy was developed for each database by using a combination of free texts and controlled vocabularies (Mesh). The search for these articles was carried out until January 2, 2024. The following search items were used (“prevalence” OR “magnitude” OR “epidemiology), AND (“poor sleep quality” OR “poor sleep” OR “sleep quality”) AND (“associated factors” OR “risk factors” OR “determinants” OR “predictors” OR “correlate”) AND (“University” OR “College” AND using African search filter developed by Pienaar et al. to identify prevalence studies (51). The Preferred Reporting Items for Systematic Reviews and Meta-Analyses (PRISMA) guidelines were followed in conducting this systematic review and meta-analysis.

### Eligibility criteria

#### Inclusion criteria

Cross-sectional studies that reported the prevalence of poor sleep quality among university students published in peer-reviewed journals in English language only, conducted in the continent of Africa were included. The tool used was the Pittsburg Sleep Quality Index only to diagnose poor sleep quality, and published from March 2011 to June 2023 were included in this review and meta-analysis.

#### Exclusion criteria

Studies were excluded if they did not report the prevalence of poor sleep quality, case reports, reviews, poster presentations, and editorial letters. Studies published in non-English languages, and conducted outside the continent of Africa were excluded. Besides this studies without access to the full data and duplicated studies were also excluded.

#### Outcome of interest

The primary outcome of this review was to determine the pooled prevalence of poor sleep quality among university students in Africa. The second outcome was to identify the pooled effects of factors associated with poor sleep quality. STATA version 14.0 was used to determine the pooled prevalence of depression, and the odds ratio (OR) was used to identify the pooled effect size of factors associated with poor sleep quality.

### Study selection and quality assessment

The articles that were retrieved were imported into EndNote X7 (Clarivate, London, UK) for gathering and arranging search results as well as eliminating duplicate entries. Three authors (GK, YAW, and GMT) evaluated the quality of the primary studies using the Joanna Briggs Institute (JBI) quality appraisal criteria. There are nine items on this quality assessment checklist, ranging from 0 to 9 (0–4 low, 5-7 medium, and 8 and above good quality (52). Those articles with high and medium quality (greater than or equal to 5) were included in the final analysis.

### Data extraction

Using a standardized data extraction format, four reviewers (FA, MM, SF, and GR) independently extracted all the necessary data from primary articles. After a careful review of the article titles, abstracts, and full texts, this was arranged using Microsoft Excel. Finally, articles approved by the four reviewers in the selection processes were included in the study, and any disagreements were resolved through discussions with other authors to reach a consensus. For instance, the first author’s name, study design, study year, publication year, country/region in which the study was conducted, a screening tool used to examine sleep quality, type of students, sample size, and prevalence of poor sleep quality were extracted. The combined estimated effects of the related covariates and prevalence of sleep quality together with their 95% confidence intervals (CIs) and odd ratios were also extracted.

### Statistical procedure

The extracted data were entered into a Microsoft Excel spreadsheet and then exported to STATA version 14.0 for analysis. The pooled prevalence of poor sleep quality along with the 95% confidence intervals was visually displayed using a forest plot. The degree of heterogeneity among the included articles was determined by the index of heterogeneity (I^2^ statistics) (53). A random-effect meta-analysis model was used to determine the pooled effect size of all the included studies due to variations of effects from individual studies. The potential sources of heterogeneity were identified using sub-group, and sensitivity analysis and meta regression. Subgroup analyses were done by using study area (Country), Region, and type of population (medical students only, health science students, and general university students). Publication bias was assessed by using both observation of the symmetry in the funnel plots and Egger weighted regression tests (54, 55). A p-value of <0.05 in Egger’s test was considered to have statistically significant publication bias.

## Results

### Identified studies

**Figure 1:**
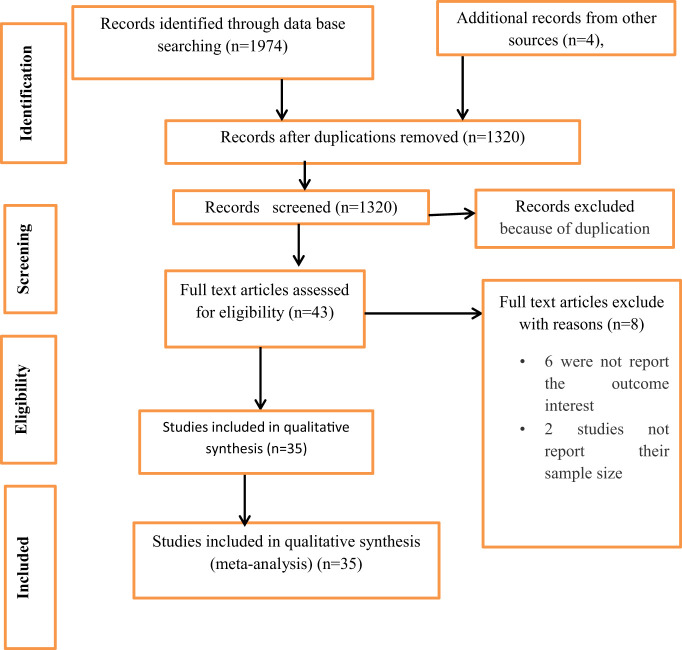
Flow charts to describe the selection of studies for the systematic review and meta-analysis on the prevalence of poor sleep quality among university students in Africa.

A total of 1978 articles were retrieved through database literature searching, including manual searching. Of these, 658 articles were excluded due to duplication, and 1277 unrelated articles were excluded by their title and abstract. The remaining 43 full-text articles were assessed for inclusion; of them, 8 full-text articles were excluded with reasons. Despite the fact that these 8 articles included a complete skeleton, the necessary information like the outcome of interest (the prevalence of poor sleep quality), and the sample size was missing. Finally, all 35 studies were included in the final meta-analysis (**Figure 1**).

### Characteristics of included studies

A total of 35 published articles from 11 countries among 16,275 university students were included in this review. All the articles included in these studies were a cross-sectional study design and the sleep quality was assessed with the Pittsburgh Sleep Quality Index. Of the 35 studies included, six studies were conducted in Nigeria, 6 in Ethiopia, 5 in Egypt, 4 in Tunisia, 3 in Ghana, 3 in Sudan, 2 in Kenya, 2 in Zambia, 2 in Morocco, 1 in Rwanda, and 1 in Libya. The study period of 25 articles were reported and conducted between March 2010 and March 2022 whereas ten studies did not report the study period. The included study was also published between March 2011 and June 2023. In terms of the study population that was involved, out of the total number of studies done, twenty-three were specifically undertaken among medical students, eight studies were conducted among general university students, and four studies were conducted among students studying health sciences (**Table 1**).

**Table 1:** Characteristics of original articles included in this systematic review and meta-analysis on poor sleep quality among university students in Africa.

Authors	Country	Study year	Publication Years	Study population	Sample size	Poor sleep quality in %
Hauwanga (56)	Kenya	Not reported	2020	General health science students	378	80
Nyamute et al. (57)	Kenya	2019	2021	Medicine	336	69.9
Lemma et al. (17)	Ethiopia	NR	2012	General university students	2551	55.8
Seyoum et al. (58)	Ethiopia	2021	2022	Medicine	224	57.6
Zeru et al., 2020 (59)	Ethiopia	2017	2020	General health science students	404	54.2
Negussie et al., 2021 (60)	Ethiopia	2019	2021	General health science students	365	60.8
Thomas and Sisay, 2019 (61)	Ethiopia	2017	2019	Medicine	372	37.2
Wondie et al., 2021 (47)	Ethiopia	2019	2021	Medicine	576	62
Akowuah et al., 2021 (62)	Ghana	2020	2021	General university students	362	62.43
Lawson et al., 2019 (63)	Ghana	2014-2015	2019	Medicine	153	56.2
Yeboah et al., 2022 (64)	Ghana	2018-2019	2022	General university students	340	54.1
James et al., 2011 (65)	Nigeria	2010	2011	Medicine	255	32.5
Seun-Fadipe and Mosaku, 2017 (66)	Nigeria	NR	2017	General university students	505	50.1
Ogunsemi et al., 2018 (67)	Nigeria		2018	General health science students	186	64
Ahmadu et al., 2022 (68)	Nigeria	2019	2022	Medicine	181	53
Awopeju et al., 2020 (69)	Nigeria	NR	2020	General university students	400	68
Seun-Fadipe and Mosaku, 2017 (70)	Nigeria	NR	2017	General university students	317	49.5
Zafar et al., 2020 (71)	Sudan		2020	Medicine	199	82.5
Mirghani et al., 2015 (72)	Sudan		2020	Medicine	140	67.9
Abdelghyoum Mahgoub and Mustafa, 2022 (73)	Sudan	2021	2022	Medicine	273	62
Mohamed and Moustafa, 2021 (74)	Egypt	2018-2019	2021	Medicine	150	58.7
Elwasify et al., 2016 (75)	Egypt	2015	2016	Medicine	1182	53.3
Dongolet al., 2022 (76)	Egypt	2020	2022	General university students	2474	79.3
Elsheikh et al., 2023 (77)	Egypt	2021-2022	2023	Medicine	1184	63.1
Ahmed Salama, 2017 (78)	Egypt	2016-2017	2017	Medicine	505	58.5
Gassara et al., 2016 (79)	Tunisia	NR	2016	Medicine	74	63.5
Maalej et al., 2018 (80)	Tunisia	2015-2016	2018	Medicine	184	80.4
Amamou et al., 2022 (81)	Tunisia	2017-2018	2022	Medicine	202	47
Saguem et al., 2022 (82)	Tunisia	2020	2022	Medicine	251	72.5
Mvula et al., 2021 (83)	Zambia		2021	General university students	212	79.2
Mwape and Mulenga, 2019 (84)	Zambia	2018	2019	Medicine	157	59.6
Hangouche et al., 2018 (85)	Morocco	2017	2018	Medicine	457	58.2
Jniene et al., 2019 (86)	Morocco	2018	2019	Medicine	286	35.3
Nsengimana et al., 2023 (87)	Rwanda	2021	2023	Medicine	290	80
El Sahly et al., 2020 (88)	Libya	2019	2020	Medicine	150	76.67

### Prevalence of poor sleep quality

**Figure 2:**
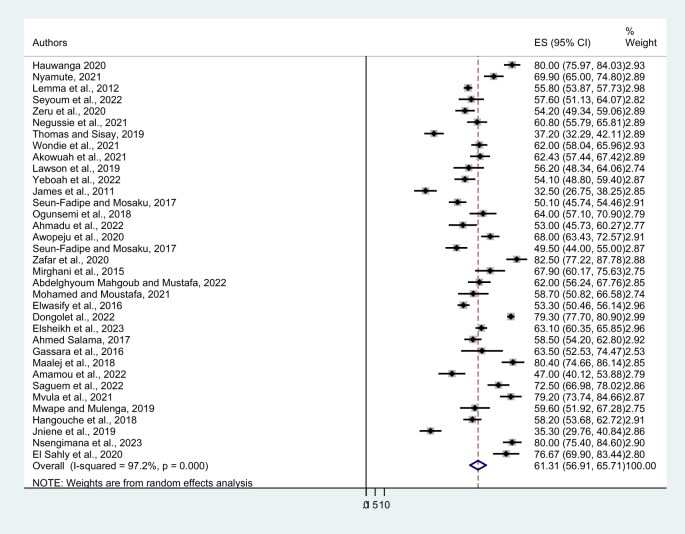
Forest plot showing the pooled prevalence of poor sleep quality among university students in Africa.

Thirty-five published articles were included in this systematic review and meta-analysis to estimate the pooled prevalence of poor sleep quality among university students. The minimum prevalence of the included study was 32.5% from Nigeria and the maximum was 82.5% from Sudan. The pooled prevalence of poor sleep quality was found to be 63.31% (95%CI: 56.91-65.71). The I^2^ test result showed higher heterogeneity (I^2 = ^97.2, P= 0.000) (**Figure 2**). Therefore, a random effect meta-analysis model was computed to estimate the pooled prevalence of poor sleep quality. To identify the possible sources of heterogeneity, different factors associated with the heterogeneity such as study areas that is countries and regions, and type of study population were investigated by using univariate meta-regression models.

### Publication bias

**Figure 3:**
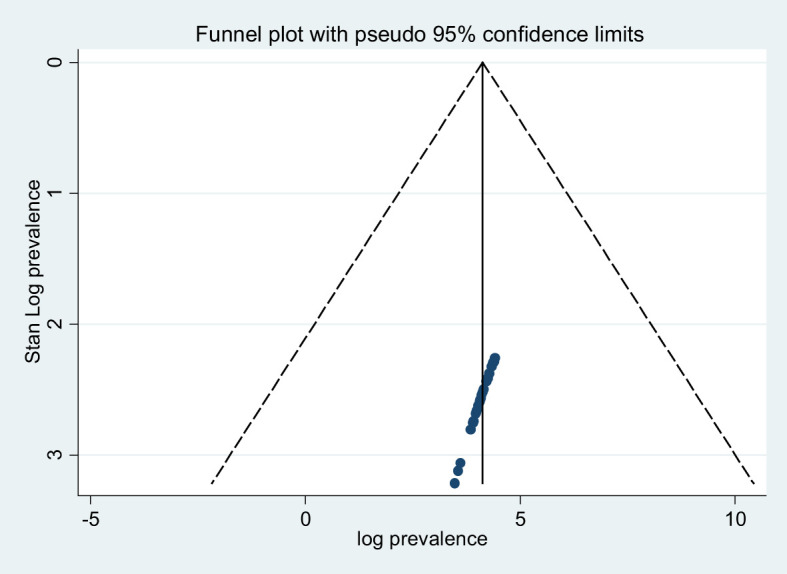
A funnel plot test of poor sleep quality among university students.

A funnel plot and Egger’s regression test were used to check the existence of potential publication bias. The result of the funnel plot triangle indicates a symmetric distribution indicating the absence of publication bias within the included studies (**Figure 3**). The Egger’s regression weighted test for publication bias revealed also no statistically significant evidence (P = 0.099) (**Table 2**).

**Table 2:** Egger’s test of poor sleep quality among university students in Africa.

Std_Eff	Coef.	Std. Err.	t	P>|t|	[95% Conf. Interval]	
slope	71.18823	4.769428	14.93	0.000	61.48476	80.8917
bias	-3.764017	2.220055	-1.7	0.099	-8.280753	0.752719

### Subgroup analysis

To identify the possible source of heterogeneity, a subgroup analysis was performed based on the region, study area (country) where studies were conducted, and the type of study population of the included study. Accordingly, the combined prevalence of poor sleep quality in East, North, West, and South Africa were 61.31 (95% CI: 56.91-65.71), 62.23 (95% CI: 54.07-70.39), 54.43 (95% CI: 47.39-61.48), and 69.59 (95% CI: 50.39-88.80) respectively. Across countries relatively high prevalence of poor sleep quality were observed in Rwanda and Libya, Kenya, and Sudan, which results in 78.95 (95% CI: 75.14-82.75), 75.05 (95% CI: 65.155-84.946), 70.89 (95% CI: 57.60-84.17) respectively and the minimum was in Morocco (46.81 (95% CI: 24.37-69.25)). A sub-group analysis based on the study population also shows that the prevalence of poor sleep quality among health science students was 64.81 (95% CI: 52.36-77.25) whereas specifically among medical students was 60.33 (95% CI: 54.96-65.69) (**Table 3**).

**Table 3:** Subgroup analysis of poor sleep quality among university students in Africa.

Characteristics	Studies	Sample	Prevalence in % (95% CI)	I^2^ (%)	p-value	Egger test
Prevalence of poor sleep quality	35	16,275	61.31 (56.91-65.71)	97.2	0.000	0.099
Region						
East Africa	12	6,108	64.15 (58.99-71.31)	89.8	0.000	
North Africa	12	7099	62.23 (54.07-70.39)	98.0	0.000	
West Africa	9	2699	54.43 (47.39-61.48)	93.0	0.000	
South Africa	2	369	69.59 (50.39-88.80)	94.0	0.000	
Country						
Nigeria	6	1,844	52.84 (42.61-63.08)	95.2	0.000	
Ethiopia	6	4,492	62.0 (58.036-65.964)	92.7	0.000	
Egypt	5	5495	65.675 (50.676-74.673)	98.8	0.000	
Tunisia	4	711	65.991 (51.169-80.813)	94.7	0.000	
Ghana	3	855	57.786 (52.22-63.35)	62.3	0.070	
Sudan	3	612	70.89 (57.60-84.17)	92.8	0.000	
Kenya	2	714	75.05 (65.155-84.946)	89.7	0.002	
Morocco	2	743	46.81 (24.37-69.25)	97.5	0.000	
Zambia	2	369	69.60 (50.39-88.80)	94.0	0.000	
Rwanda and Libya	2	440	78.95 (75.14-82.75)	0.425	0.0	
Study population						
General University students	8	7161	62.34 (52.42-72.25)	98.6	0.000	
General Health science students	4	1333	64.81 (52.36-77.25)	95.9	0.000	
Medical students only	23	7781	60.33 (54.96-65.69)	96.0	0.000	

### Sensitivity analysis

The sensitivity analysis was conducted to examine the heterogeneity of those studies and determine the impact of each study’s findings on the overall prevalence of poor sleep quality. The result showed that all values are within the estimated 95% CI, indicating that the omission of one study had no significant impact on the prevalence of this meta-analysis (**Table 4**).

**Table 4:** Sensitivity analysis of poor sleep quality among university students in Africa.

Study omitted	Estimate 95% CI	Heterogeneity	
		I ^2^ (%)	P value
Hauwanga 2020 (56)	60.75 (56.32-65.18)	97.1	0.000
Nyamute, 2021 (57)	61.05 (56.54-65.56)	97.3	0.000
Lemma et al., 2012 (35)	61.48 (56.84-66.12)	97.1	0.000
Seyoum et al., 2022 (58)	61.42 (56.93-65.91)	97.3	0.000
Zeru et al., 2020 (59)	61.52 (57.03-66.02)	97.02	0.000
Negussie et al., 2021 (60)	61.33 (56.81-65.84)	97.3	0.000
Thomas and Sisay, 2019 (61)	62.03 (57.73-66.33)	97.0	0.000
Wondie et al., 2021 (47)	61.29 (56.74-65.4)	97.3	0.000
Akowuah et al., 2021 (62)	61.28 (56.76-65.80)	97.3	0.000
Lawson et al., 2019 (63)	61.45 (56.98-65.93)	97.3	0.000
Yeboah et al., 2022 (64)	61.52 (57.04-66.01)	97.2	0.000
James et al., 2011 (65)	62.16 (57.87-66.45)	97.0	0.000
Seun-Fadipe and Mosaku, 2017 (66)	61.65 (57,19-66.11)	97.2	0.000
Ogunsemi et al., 2018 (67)	61.23 (56.74-65.73)	97.3	0.000
Ahmadu et al., 2022 (68)	61.55 (57.07-66.02)	97.3	0.000
Awopeju et al., 2020 (69)	61.11 (56.59-65.63)	97.3	0.000
Seun-Fadipe and Mosaku, 2017 (70)	61.66 (57.20-66.12)	97.2	.0.000
Zafar et al., 2020 (71)	60.68 (56.26-65.11)	97.2	0.000
Mirghani et al., 2015 (72)	61.12 (56.64-65.61)	97.3	0.000
Abdelghyoum Mahgoub and Mustafa, 2022 (73)	61.29 (56.78-65.80)	97.3	0.000
Mohamed and Moustafa, 2021 (74)	61.38 (56.90-65.87)	97.3	0.000
Elwasify et al., 2016 (75)	61.55 (57.04-66.07)	97.1	0.000
Dongol et al., 2022 (76)	60.76 (56.82-64.69)	95.7	0.000
Elsheikh et al., 2023 (77)	61.25 (56.62-65.89)	97.3	0.000
Ahmed Salama, 2017 (78)	61.39 (56.87-65.92)	97.3	0.000
Gassara et al., 2016 (79)	61.25 (56.78-65.72)	97.3	0.000
Maalej et al., 2018 (80)	60.75 (56.30-65.200	97.2	0.000
Amamou et al., 2022 (81)	61.72 (57.27-66.17)	97.2	0.000
Saguem et al., 2022 (82)	60.98 (56.49-65.47)	97.3	0.000
Mvula et al., 2021 (83)	60.78 956.33-65.24)	97.2	0.000
Mwape and Mulenga, 2019 (84)	61.36 (56.87-65.84)	97.3	0.000
Hangouche et al., 201 (85)	61.40 (56.88-65.92)	97.3	0.000
Jniene et al., 2019 (86)	62.08 (57.77-66.39)	97.0	0.000
Nsengimana et al., 2023 (87)	60.75 (56.31-65.19)	97.2	0.000
El Sahly et al., 2020 (88)	60.87 (56.40-65.34)	97.2	0.000

### Meta regression

In this study, meta-regression was done on continuous covariates including years of publication, type of study populations (general university students, general health science students, and medical students only), sample size, and countries. The results showed that only publication year (p = 0.021) was a source of heterogeneity for this study. However, the pooled prevalence of poor sleep quality was not associated with sample size (p = 0.772), countries (p = 0.24), and types of study populations (p = 0.896) (**Table 5**).

**Table 5:** Meta regression of poor sleep quality among university students in Africa.

Variable	Coefficient	P-value
Publication year	1.65	0.021
Sample size	0.016	0.772
Country	1.045	0.24
Types of study population	0.50	0.896

### Associated factors of poor sleep quality among university students

**Figure 4:**
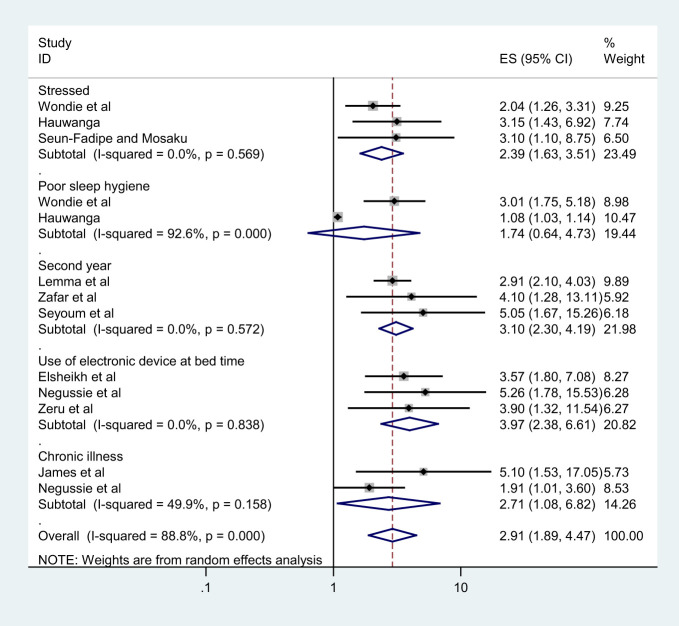
The forest plot shows associated factors of poor sleep quality among university students in Africa.

From the included primary studies, there are different factors associated with poor sleep quality among university students, but we include those reported in more than one study. For instance, being stressed, having poor sleep hygiene, second year, using electronic devices at bedtime, and having comorbid chronic illness were factors reported and associated with poor sleep quality among university students more than once. The result of this meta-analysis indicated that being stressed is 2.4 times more likely to have poor sleep quality than not being stressed (AOR= 2.39; 95% CI: 1.63 to 3.51). The pooled odds ratio (AOR) demonstrated that the odds of poor sleep quality were 3.1 higher in participants who were in the second academic year (AOR= 3.10; 95% CI: 2.30 to 4.19) than students in other academic years. In addition, participants who use electronic devices at bedtime (AOR= 3.97 95% CI: 2.38 to 6.61) were nearly 4 times to have poor sleep quality than their counterparts. The current meta-analysis also shows that having a comorbid chronic illness was about 2.7 (AOR = 2.71; 95% CI: 1.08, 6.82) times more likely to have poor sleep quality than students who have not (**Figure 4**).

## Discussion

Sleep is an important physiological process for humans. University students in African countries often report poor quality of Sleep due to changing social opportunities and increasing academic demands. However, the results of poor sleep quality among university students across nations and in between different studies vary. This systematic review and meta-analysis of 35 studies aimed to estimate the pooled prevalence and associated factors of poor sleep quality among university students in 11 African countries.

In this meta-analysis and systematic review, the pooled prevalence of poor sleep quality was found to be 63.31% with a 95% CI (56.91-65.71). This result is in line with findings from other studies. According to a systematic review and meta-analysis, which was done to determine the global prevalence rate of poor sleep quality among university students, the result was 57% (89). The current finding is also comparable with a systematic review and a meta-analysis study conducted among Korean University Students yielded 59.2% (90). A global systematic and meta-analysis study of the general population found that 57.3% of respondents had poor sleep quality, which was consistent with the findings of the current study (91).The results of a systematic review of twelve studies among Indian university students range from 25 to 72%, which is consistent with the findings of the current meta-analysis on poor sleep quality among students in Africa (92).

On the contrary, the current finding was significantly higher than with a different systematic review and meta-analysis study that was carried out at a different period. For instance, in a global systematic review and meta-analysis study conducted on sleep disruption in medicine students and its relationship with impaired academic performance, 39.8% of them reported having poor sleep quality (93). The prevalence of poor sleep quality was 51.45% in the global systematic review and meta-analysis study on the prevalence of sleep problems among 59427 medical students from 109 Studies (19). However, the sub-group analysis in the current meta-analysis studies showed that the prevalence of poor sleep quality, specifically among African medical students was 60.33 (95% CI: 54.96-65.69). In a comprehensive meta-analysis of observational studies among 24,884 medical students across 50 studies, the prevalence of poor sleep quality was 52.7% (94). The current finding is also higher than the results of previous meta-analyses that were carried out in 28 different countries, with results of 9.6% (95), 55.6% in a global meta-analysis (19), 53% in the Ethiopian population (96), 51.0% in Brazil (97), 24.1% in China (98), and 43.4% in US college students (99). A review of the causes of poor sleep quality in African young dults indicates that poor sleep quality is higher in Africa than other continents (45). When young African people do manage to get into committed partnerships, they often wind up spending a large portion of their evenings on social media (100). Some of them may indulge in drug and alcohol abuse with several partners, frequent clubs, or spend a significant portion of their sleep hours in sexual activities (100, 101). Because of this, most young adults may suffer from sleep deprivation and excessive daytime sleepiness as their bodies attempt to harmonize their naturally delayed schedule with their daily social schedules and activities at school (102). The other possible reason could be the impact that an individual’s race or ethnicity has on the quality of their sleep. It follows that mental health concerns and sleep disturbances are related health problems. According to two studies, black people are more likely to have sleep problems, which raises the likelihood of poor sleep quality in Africa (45, 103, 104).

Poor sleep quality among university students is also higher in Africa than in other studies because of the different genes involved in sleep activity (105–107), and the immune system (108–110) that Africans have. The other explanation could be because health care providers, insurance companies, governments, and the general population in Africa have little knowledge about sleep disorders and the grave repercussions they can have. One of the main problems in many African countries is the state of the health and insurance systems. The low socioeconomic level is frequently accompanied by a lack of sleep labs, clinics, and diagnostic equipment as well as a high cost of medications (111, 112).

However, the current finding is lower than a multinational cross-sectional study involving medical students during the COVID-19 pandemic, which found that 73.5% of students had poor sleep quality (20). The COVID-19 pandemic has led to increased stress, anxiety, and psychological distress among students, potentially affecting their sleep quality. The restrictions and fear of infection also led to a negative mood, which can negatively impact sleep quality. The virus primarily targets the respiratory system, and sudden outbreaks, rising death counts, and social disruptions have contributed to a decline in sleep quality. The pandemic indirectly affects college students’ moods and sleep quality (113–117).

Regarding factors affecting poor sleep quality among university students, three of the included studies in this meta-analysis study disclosed that students who have been stressed were more likely to have poor sleep quality as compared with non-stressed. The pooled result of this meta-analysis indicated that stressed students were about 1.4 times more likely to have poor sleep quality as compared to their counterparts. University students’ various activities and stressors, including studying during the night, can lead to poor sleep quality due to psychological distress (118). Compared to other students, especially medical students, they experience stress more frequently. Medical students face a stressful environment due to academic requirements and workload. They often reduce sleep to cope with the demands, leading to poor mental and physical health. Factors such as on-call duties, disease contact, and examinations contribute to this stress. Consequently, they may not prioritize sleep, leading to poor sleeping quality (34, 119). Stress plays a big role in how well people sleep, and many stresses from daily lifeare associated with poor sleep. For example, a longitudinal study of people with good sleep quality at baseline found that the most significant predictors of disrupted sleep at follow-up were daytime stress level and nighttime worries (120). Similar to this, as measured by polysomnography, healthy volunteers who experienced higher levels of stress at work had substantially more fragmented sleep and lower sleep efficiency (121). Chronic activation of stress responses, such as the sympathetic-adrenal-medullary axis and hypothalamic-pituitary-adrenal axis, can produce epinephrine and cortisol, known as stress hormones. This stress hormone has a negative effect on students sleep quality and their academic performances (118, 122). Uncontrollable worries about stress events trigger emotional arousal, leading to cognitive biases and distorted evaluations, resulting in subjective sleep quality decline (123–126).

In this meta-analysis, the year of study is also one of the factors contributing to poor sleep quality among university students. The year 2 students were more than 3 times to have poor sleep quality compared with students in other academic years. Several other investigations also discovered that the distribution of sleep quality varied across the years of study. A study in Saudi Arabian and Brazilian students showed that the odds of having poor sleep were significantly higher among second and fourth-year students (127, 128). However, according to a study in Greece, sixth-year medical students were more odds to have poor sleep quality than other students (129). A study conducted at a Chinese university revealed that fifth-year students were more negatively impacted by sleep deprivation (130), while other studies found no differences in the general quality of sleep by academic years (131). This variety may be the result of variations in the curriculum used in different countries and universities, as well as differences in social and academic demands (58, 132).

University students who use electronic devices at bedtime had approximately four times higher rates of poor sleep quality than their counterparts. This study is supported by a global meta-analysis study in which using electronic devices like smartphones was associated with poor sleep quality (133). Excessive use of electronic devices is significantly associated with poor sleep quality, according to two further global meta-analyses, one of which was conducted on medical students and the other on adolescents (89, 134). The quality of sleep is greatly impacted by using electronic devices before and during bedtime, including computers, music players, televisions, social networking sites, and cell phones. Intimate relationship-seeking young adults in Africa often find themselves interacting with their partners on mobile social media platforms like Facebook, Instagram, Snapchat, WhatsApp, and TikTok late at night, before going to bed early the next day (135, 136). Regular use of such material thus increases the likelihood of prolonged sleep onset, short duration, and extended start latency (136–138). There are physiological changes in students’ circadian rhythm and homeostatic sleep patterns. Because of this, the majority of young adults may experience sleep deprivation and excessive fatigue during the day as their bodies try to adjust to their naturally delayed schedule in order to fit in with their everyday social routines and academic obligations (102, 139). Researchers have hypothesized that using mobile devices also affects the quality of one’s sleep through a variety of mechanisms, including electromagnetic fields emitted by the device, which change melatonin rhythms, cerebral blood flow, and other related brain activities recorded in waking electroencephalograms (136, 140–142).

In this systematic review and meta-analysis study, a substantial association between having comorbid medical illness and poor sleep quality was also found. The prevalence of poor sleep quality among students was 2.7 times more common among students who had comorbid medical illnesses than those who did not. This was supported by a worldwide investigation involving seven nations as well as a US study on multi-campus students (20, 99). Poor sleep quality can also be brought on by the stress of a chronic condition. Heartburn, which is brought on by stomach acid backing up into the esophagus, is frequently linked to trouble sleeping. Uncontrolled diabetes can also contribute to trouble sleeping through night sweats, and frequent urination. Students with heart failure may experience dyspnea when they wake up in the middle of the night. People with arthritis pain may find it difficult to go off to sleep. Furthermore, a variety of over-the-counter and prescription drugs used to address these and other health issues might lower the quality of sleep (143–145).

## Strengths and limitations of the study

The study’s strength lies in its pooled effect of multiple studies (35 articles) and large sample size of 16,275 university students. Additionally, we included articles from all regions of Africa (North, South, East, and West) to help generalize the findings throughout the continent. The study’s limitations include the fact that the age range was not adequately defined in the primary studies included in the review and meta-analysis and that only English-language publications were taken into consideration because of language bias. This review revealed significant between-study heterogeneity. Other than the ones currently listed, there may be more factors contributing to heterogeneity.

## Conclusion and recommendations

According to this study, there is a high pooled prevalence of poor sleep quality among university students in Africa. Being stressed, using electronic devices in bed, being a second year, and having concomitant medical conditions were all associated with poor sleep quality. Thus, early detection and adequate intervention are important for improving sleep quality among university students. The establishment of academic counseling centers with an emphasis on improving sleep quality, bolstering students’ study skills, and helping them cope with their stressful surroundings is advised for the management of the sleep quality of university students. University students can also benefit from improved physical health and reduced stress levels to get better sleep. Students are also recommended to limit their use of electronic devices, such as smartphones, right before bed.

## Data availability statement

The original contributions presented in the study are included in the article/**Supplementary Material**. Further inquiries can be directed to the corresponding author.

## Author contributions

GN: Conceptualization, Formal analysis, Investigation, Methodology, Project administration, Supervision, Writing – original draft, Writing – review & editing. GMT: Data curation, Formal analysis, Investigation, Methodology, Writing – original draft, Writing – review & editing. GR: Methodology, Writing – original draft, Writing – review & editing. FA: Data curation, Investigation, Methodology, Writing – original draft, Writing – review & editing. TT: Methodology, Writing – original draft. MAK: Data curation, Investigation, Writing – original draft. GT: Formal analysis, Writing – original draft, Writing – review & editing. SF: Formal analysis, Methodology, Writing – original draft, Writing – review & editing. YW: Formal analysis, Methodology, Writing – original draft, Writing – review & editing. TS: Formal analysis, Methodology, Writing – original draft. GK: Formal analysis, Investigation, Writing – original draft, Writing – review & editing. MM: Formal analysis, Investigation, Methodology, Writing – original draft, Writing – review & editing.
