# Supplementary material for: Sleep quality and associated factors among university students in Africa: a systematic review and meta-analysis study
Source: Front Psychiatry. 2024 Mar 11;15:1370757. doi: 10.3389/fpsyt.2024.1370757 (PMC10979362; doi:10.3389/fpsyt.2024.1370757)
Supplement: Supplementary file 2 [file Table_2.docx]

Additional file 2: Quality assessment of original articles studies on depression and its associated factors among IDPs in Sub Saharan region.

| Author, year of  Publication | Q1 | Q2 | Q3 | Q4 | Q5 | Q6 | Q7 | Q8 | Q9 | Total score (9%) |
| --- | --- | --- | --- | --- | --- | --- | --- | --- | --- | --- |
| Hauwanga 2020 | Y | Y | Y | Y | Y | Y | Y | Y | Y | 9 |
| Nyamute, 2021 | Y | Y | Y | Y | Y | Y | Y | Y | Y | 9 |
| Lemma et al., 2012 | Y | Y | Y | Y | Y | Y | Y | Y | Y | 9 |
| Seyoum et al., 2022 | Y | Y | Y | Y | NA | Y | Y | NA | Y | 7 |
| Zeru et al., 2020 | Y | Y | Y | Y | Y | NA | NA | Y | Y | 7 |
| Negussie et al., 2021 | Y | Y | NA | Y | Y | Y | Y | Y | Y | 8 |
| Thomas and Sisay, 2019 | Y | Y | Y | Y | Y | Y | Y | Y | Y | 9 |
| Wondie et al., 2021 | Y | Y | NA | Y | Y | Y | NA | Y | Y | 7 |
| Akowuah et al., 2021 | Y | NA | NA | Y | Y | NA | Y | NA | Y | 6 |
| Lawson et al., 2019 | Y | Y | Y | Y | Y | Y | NR | Y | Y | 8 |
| Yeboah et al., 2022 | Y | Y | Y | Y | Y | Y | Y | Y | Y | 9 |
| James et al., 2011 | Y | NA | Y | Y | Y | Y | Y | Y | Y | 8 |
| Seun-Fadipe and Mosaku, 2017 | Y | Y | Y | Y | NA | Y | NA | Y | Y | 7 |
| Ogunsemi et al., 2018 | Y | Y | Y | NA | Y | NA | Y | Y | Y | 7 |
| Ahmadu et al., 2022 | Y | Y | Y | Y | Y | Y | Y | Y | Y | 9 |
| Awopeju et al., 2020 | Y | Y | NA | Y | Y | Y |  | Y | Y | 8 |
| Seun-Fadipe and Mosaku, 2017 | Y | Y | Y | Y | Y | Y | NR | Y | Y | 8 |
| Zafar et al., 2020 | Y | Y | Y | Y | Y | Y |  | NA | Y | 8 |
| Mirghani et al., 2015 | Y | Y | NA | Y | NA | Y | Y | Y | Y | 7 |
| Abdelghyoum Mahgoub and Mustafa, 2022 | Y | Y | Y | Y | Y | Y | Y | Y | NA | 8 |
| Mohamed and Moustafa, 2021 | Y | Y | Y | Y | Y | Y | Y | Y | Y | 9 |
| Elwasify et al., 2016 | Y | Y | Y | Y | Y | Y | Y | Y | Y | 9 |
| Dongolet al., 2022 | Y | Y | Y | Y | Y | Y | Y | NR | Y | 8 |
| Elsheikh et al., 2023 | Y | Y | NR | Y | Y | Y | NA | Y | Y | 7 |
| Ahmed Salama, 2017 | Y | Y | Y | Y | Y | Y | NA | Y | Y | 8 |
| Gassara et al., 2016 | Y | NA | Y | Y | Y | Y | Y | Y | Y | 8 |
| Maalej et al., 2018 | Y | Y | Y | Y | Y | Y | Y | NA | Y | 8 |
| Amamou et al., 2022 | Y | Y | Y | Y | Y | Y | Y | Y | Y | 9 |
| Saguem et al., 2022 | Y | Y | Y | Y | Y | Y | Y | Y | NA | 8 |
| Mvula et al., 2021 | Y | Y | Y | Y | Y | Y | Y | Y | Y | 9 |
| Mwape and Mulenga, 2019 | Y | Y | Y | Y | Y | Y | Y | NA | Y | 8 |
| Hangouche et al., 2018 | Y | Y | Y | NA | NA | NA | Y | Y | Y | 6 |
| Jniene et al., 2019 | Y | Y | Y | Y | Y | Y | Y | Y | NA | 8 |
| Nsengimana et al., 2023 | Y | Y | Y | Y | Y | Y | NA | Y | Y | 8 |
| El Sahly et al., 2020 | Y | Y | Y | Y | Y | Y | Y | Y | Y | 9 |

**Key:** **Y**= Yes; **NR**= Not reported, **NA**=Not appropriate

**Question codes:**

1. Was the sample frame appropriate to address the target population?

2. Were study participants sampled in an appropriate way?

3. Was the sample size adequate?

4. Were the study subjects and the setting described in detail?

5. Was the data analysis conducted with sufficient coverage of the identified sample?

6. Were valid methods used for the identification of the condition?

7. Was the condition measured in a standard, reliable way for all participants?

8. Was there appropriate statistical analysis?

9. was the response rate adequate, and if not, was the low response rate managed appropriately?
